# Multiswarm comprehensive learning particle swarm optimization for solving multiobjective optimization problems

**DOI:** 10.1371/journal.pone.0172033

**Published:** 2017-02-13

**Authors:** Xiang Yu, Xueqing Zhang

**Affiliations:** 1 Provincial Key Laboratory for Water Information Cooperative Sensing and Intelligent Processing, Nanchang Institute of Technology, Nanchang, Jiangxi, China; 2 Department of Civil and Environmental Engineering, The Hong Kong University of Science and Technology, Hong Kong; Beihang University, CHINA

## Abstract

Comprehensive learning particle swarm optimization (CLPSO) is a powerful state-of-the-art single-objective metaheuristic. Extending from CLPSO, this paper proposes multiswarm CLPSO (MSCLPSO) for multiobjective optimization. MSCLPSO involves multiple swarms, with each swarm associated with a separate original objective. Each particle’s personal best position is determined just according to the corresponding single objective. Elitists are stored externally. MSCLPSO differs from existing multiobjective particle swarm optimizers in three aspects. First, each swarm focuses on optimizing the associated objective using CLPSO, without learning from the elitists or any other swarm. Second, mutation is applied to the elitists and the mutation strategy appropriately exploits the personal best positions and elitists. Third, a modified differential evolution (DE) strategy is applied to some extreme and least crowded elitists. The DE strategy updates an elitist based on the differences of the elitists. The personal best positions carry useful information about the Pareto set, and the mutation and DE strategies help MSCLPSO discover the true Pareto front. Experiments conducted on various benchmark problems demonstrate that MSCLPSO can find nondominated solutions distributed reasonably over the true Pareto front in a single run.

## 1. Introduction

Multiobjective optimization deals with multiple objectives that often conflict with each other. The presence of such multiple objectives gives rise to a set of nondominated solutions. Multiobjective optimization methods are either generating or preferences-based [1]. Regarding the generating methods, no preferences of the objectives are given, and nondominated solutions reasonably covering the entire extension of the true Pareto front need to be found so as to provide the decision maker diverse information to determine the final tradeoff [2]. The preferences-based methods, with the preferences of the objectives known in advance, convert the multiple objectives into a single objective through techniques such as weighting and *ε*-constraint; the single-objective problem can then be solved using a single-objective optimizer. It has been noted in [3] that the weighting technique cannot find a nondominated solution on the nonconvex portions of the Pareto front and the *ε*-constraint technique finds a nondominated solution only if certain conditions are satisfied. The Pareto dominance relationship doesn’t rely on any preferences knowledge and can be used in the generating methods to handle the multiple objectives directly.

Over the past several decades, a number of generating multiobjective metaheuristics (MOMHs) have been applied to solve real-world multiobjective optimization problems (MOPs) in a wide range of areas. Compared with traditional optimizers such as linear programming, nonlinear programming, optimal control theory, and dynamic programming, MOMHs are significantly more flexible as they don’t require the objectives and constraints to be continuous, differentiable, linear, or convex, and MOMHs are rather efficient. In addition, population-based MOMHs using a population of individuals (with each individual representing a candidate solution) facilitate the discovery of multiple nondominated solutions in a single run.

This paper aims to propose a high performance MOMH based on particle swarm optimization (PSO). PSO is a swarm intelligence inspired metaheuristic introduced in 1995 [4, 5]. PSO is population-based and solves a single-objective optimization problem (SOP) using a swarm of particles. All the particles “fly” in the search space. Each particle, denoted as *i*, is associated with a position, a velocity, and a fitness that indicates its performance. PSO relies on iterative learning to find the optimum. In each iteration (or generation), *i* adjusts its velocity according to its previous velocity, its historical best position (i.e. personal best position), and the personal best positions of its neighborhood particles. As indicated by the reported experimental results of some recently proposed PSO variants such as comprehensive learning PSO (CLPSO) [6], orthogonal learning PSO (OLPSO) [7], selectively informed PSO (SIPSO) [8], and PSO with limited information (LIPSO) [9], the personal best positions of *i*’s neighborhood particles need to be nontrivially and appropriately leveraged during the update of *i*’s flight trajectory so as to achieve satisfactory exploration performance on multimodal SOPs. CLPSO and OLPSO encourage *i* to learn from different exemplars (i.e. *i*’s personal best position or a position determined from *i*’s neighborhood) on different dimensions. For SIPSO, the particles take different learning strategies based on the degree of connections; a densely-connected hub particle gets full information from all the neighbors while a non-hub particle with few connections only follows the best-performed neighbor. LIPSO adjusts *i*’s velocity through the use of limited yet adequate search experience information regarding *i*’s neighborhood. PSO can handle large scale SOPs with the aid of parallelization [10].

When extending PSO to the domain of multiobjective optimization, elitists need to be stored externally [11–14] or internally [15–18]. An elitist is a solution nondominated among all the candidate solutions generated so far. Existing MOMHs either treat the outstanding MOP as a whole or involve decomposition. For multiobjective PSOs (MOPSOs) that treat the MOP as a whole [12, 13], *i*’s personal best position is determined based on Pareto dominance. An external repository stores elitists. *i* learns from its (and other particles’) personal best position(s) and an elitist selected from the external repository. Decomposition based MOPSOs decompose the MOP into multiple different SOPs. Multiple swarms/particles are used, with each swarm/particle independently optimizing a separate SOP. *i*’s personal best position is thus determined according to the corresponding single objective. The multiple swarms/particles collaborate to derive nondominated solutions through direct and/or indirect information exchange. Vector evaluated PSO (VEPSO) [11] and coevolutionary multiswarm PSO (CMPSO) [14] take advantage of multiple swarms, with each swarm focusing on optimizing a separate objective of the original multiple objectives. In VEPSO, *i* learns from its personal best position and the search experience of its neighboring swarms. In CMPSO, the swarms don’t exchange information directly, but instead the personal best position and an elitist randomly selected from the external repository are used to update *i*’s velocity. The external repository is shared by all the swarms. Multiobjective evolutionary algorithm based on decomposition (MOEA/D) [19] is a framework that lets each individual to optimize a separate SOP. Each single objective is attained using aggregation techniques such as weighted sum, Tchebycheff, and boundary intersection. Each individual evolves based on its personal search experience and its neighboring individuals’ search experience. The works [15–18] are MOPSOs based on the MOEA/D framework.

Extending from the powerful single-objective PSO variant CLPSO, this paper proposes multiswarm CLPSO (MSCLPSO) for multiobjective optimization. CLPSO has been extended to handle multiobjective optimization in [13, 20]. No decomposition is involved in multiobjective CLPSO (MOCLPSO) and attributed MOCLPSO (A-MOCLPSO) respectively proposed in [13] and [20]. MSCLPSO is the same with CMPSO [14] in terms of using multiple swarms, the way of determining the personal best position, and storing elitists in a shared external repository. MSCLPSO promotes the diversity of the elitists through respectively the crowding distance technique [21] for two-objective MOPs and the *M*-nearest-neighbors product-based vicinity distance technique for MOPs with more than two objectives [22]. The crowding distance technique works excellently in the case of two objectives, but it fails to effectively approximate the diversity of the elitists when the number of objectives is three or more [22]. MSCLPSO is novel in three aspects. First, each swarm focuses on optimizing the associated SOP strictly using CLPSO, without learning from the elitists and any other swarm. Second, mutation is applied to the elitists and the mutation strategy appropriately exploits the personal best positions and elitists. Third, a modified differential evolution (DE) strategy is applied to some extreme and least crowded elitists. The DE strategy updates an elitist based on the differences of the elitists. The personal best positions carry useful information about the Pareto set, and the mutation and DE strategies help MSCLPSO discover the true Pareto front. MSCLPSO takes the decomposition based multiswarm architecture and updates each particle *i*’s velocity purely based on the search experience of the particles in *i*’s host swarm because information determined based on Pareto dominance or some other single objective might not contribute to the optimization on *i*’s associated objective. MSCLPSO was applied to the 2-objective sustainable operation of China’s Three Gorges cascaded hydropower system in [23]. This paper gives a detailed description of MSCLPSO and presents the algorithm’s performance on a variety of benchmark MOPs.

The rest of this paper is organized as follows. In Section 2, the working principle of CLPSO, definitions related to multiobjective optimization, and a brief literature review on MOMHs are presented. Section 3 details the implementation of MSCLPSO. In Section 4, the performance of MSCLPSO is evaluated on some 2- and 3-objective benchmark MOPs. Section 5 concludes the paper.

## 2. Background

### 2.1 Comprehensive learning particle swarm optimization

Let there be *D* decision variables, the swarm of *N* particles fly in a *D*-dimensional search space. Each particle *i* (1 ≤ *i* ≤ *N*) is associated with a position *P*_*i*_ = (*P*_*i*,1_, *P*_*i*,2_, …, *P*_*i*,*D*_) and a velocity *V*_*i*_ = (*V*_*i*,1_, *V*_*i*,2_, …, *V*_*i*,*D*_). *V*_*i*_ and *P*_*i*_ are initialized randomly. In each generation, *V*_*i*_ and *P*_*i*_ are updated as follows.

$$V_{i,d}=wV_{i,d}+cr_{d}(E_{i,d}-P_{i,d})$$ (1)

$$P_{i,d}=P_{i,d}+V_{i,d}$$ (2)

where *d* (1 ≤ *d* ≤ *D*) is the dimension index; *w* is the inertia weight; *c* is the acceleration coefficient and *c* is suggested to be 1.5 [6]; *r*_*d*_ is a random number uniformly distributed in the range [0, 1]; and *E*_*i*_ = (*E*_*i*,1_, *E*_*i*,2_, …, *E*_*i*,*D*_) is the guidance vector of exemplars.

The inertia weight *w* linearly decreases. Specifically, let *k*_max_ be the predefined maximum number of generations, in each generation *k*, *w* is updated according to Eq (3).

$$w=w_{\text{max}}-(w_{\text{max}}-w_{\text{min}})\frac{k}{k_{\text{max}}}$$ (3)

where *w*_max_ and *w*_min_ are respectively the maximum and minimum inertia weights. The recommended values for *w*_max_ and *w*_min_ are respectively 0.9 and 0.4 [6].

The dimensional velocity *V*_*i*,*d*_ is usually clamped to a positive value $V_{d}^{\text{max}}$. If $V_{i,d}>V_{d}^{\text{max}}$, then *V*_*i*,*d*_ is set to $V_{d}^{\text{max}}$; or if $V_{i,d}<-V_{d}^{\text{max}}$, then *V*_*i*,*d*_ is set to $-V_{d}^{\text{max}}$. Let $\underline{P_{d}}$ and $\bar{P_{d}}$ respectively be the lower and upper bounds of the search space on dimension *d*, $V_{d}^{\text{max}}$ is suggested to be set as 20% of $\bar{P_{d}}-\underline{P_{d}}$ [6].

Let *B*_*i*_ = (*B*_*i*,1_, *B*_*i*,2_, …, *B*_*i*,*D*_) be the personal best position of *i*. After the position *P*_*i*_ is updated, *P*_*i*_ is evaluated and will replace *B*_*i*_ if *P*_*i*_ has a better fitness value.

The exemplar *E*_*i*,*d*_ can be *B*_*i*,*d*_ or *B*_*j*,*d*_ with *j* ≠ *i*. The decision to learn whether from *B*_*i*,*d*_ or *B*_*j*,*d*_ depends on a learning probability *L*_*i*_. For dimension *d*, a random number uniformly distributed in the range [0, 1] is generated. If the generated number is no less than *L*_*i*_, *i* will learn from *B*_*i*,*d*_ on dimension *d*; otherwise from *B*_*j*,*d*_. The particle *j* is selected from a 2-tournament procedure. If *E*_*i*_ happen to be the same as *B*_*i*_, ECLPSO will randomly choose one dimension to learn from some other particle’s corresponding dimensional personal best position.

An empirical expression is developed in CLPSO to set the learning probability *L*_*i*_ for each particle *i*.

$$L_{i}=0.05+0.45\frac{\text{exp}(\frac{10(i-1)}{N-1})-1}{\text{exp}(10)-1}$$ (4)

CLPSO allows each particle *i* to learn from the same exemplars until *i*’s fitness values cease improving for a refreshing gap of *h* consecutive generations. *h* is suggested to be 7 [6].

CLPSO calculates the fitness value of a particle *i* only if *i* is feasible (i.e. within $\left[\underline{P_{d}},\bar{P_{d}}\right]$ on each dimension *d*).

### 2.2 Multiobjective optimization and pareto dominance

Without loss of generality, consider a multiobjective minimization problem described in Eq (5).

$$\begin{array}{l} \text{Min }f(x)=(f_{1}(x),f_{2}(x),\ldots ,f_{M}(x))\\ \text{s}.\text{t}.\;g(x)\leq 0 \end{array}$$ (5)

where *x* = (*x*_1_, *x*_2_, …, *x*_*D*_) is the decision vector; *M* (*M* ≥ 2) is the number of objectives; *f*_*m*_ is the function or numerical simulation procedure used to evaluate the fitness of *x* on objective *m*, *m* = 1, 2, …, *M*; and *g* is the combination of constraints. Some definitions related to multiobjective optimization and Pareto dominance are given below.

Definition 1: The **search space** is **SS** = {*x* ∈ **R**^*D*^ | *g*{*x*} ≤ 0}.

Definition 2: The **objective space** is OS = {*f*(*x*) ∈ **R**^*M*^ | *x* ∈ **SS**}.

Definition 3: Given two points *a* = (*a*_1_, *a*_2_, …, *a*_*M*_) and *b* = (*b*_1_, *b*_2_, …, *b*_*M*_) in the objective space **OS**, *b* **dominate**s *a* if *b*_*m*_ ≤ *a*_*m*_ for all *m* = 1, 2, …, *M*, and *b* ≠ *a*.

Definition 4: A point *a* in the objective space **OS** is **nondominated** if there is no other point *b* in **OS** such that *b* dominates *a*.

Definition 5: A point *x* in the search space **SS** is **Pareto-optimal** if *f*(*x*) is nondominated.

Definition 6: The **Pareto set** is **PS** = {*x* ∈ **SS** | *x* is Pareto-optimal}.

Definition 7: The **Pareto front** is **PF** = {*f*(*x*) ∈ **OS** | *x*∈ **PS**}.

The objective space **OS** is partially ordered in the sense that two arbitrary points are related to each other in two possibly ways: either one dominates the other or neither dominates.

Definition 3 can be modified based on the concept of *ε*-dominance [24].

Definition 8: Given two points *a* = (*a*_1_, *a*_2_, …, *a*_*M*_) and *b* = (*b*_1_, *b*_2_, …, *b*_*M*_) in the objective space **OS**, *b* ***ε*-dominate**s *a* if *b*_*m*_—*a*_*m*_ ≤ *ε* for all *m* = 1, 2, …, *M*, and there exists one *m* such that *b*_*m*_—*a*_*m*_ < *ε*, where *ε* is a predefined small positive number.

### 2.3 Brief literature review on MOMHs

MOMHs have attracted extensive research interests. A large number of MOMHs have been proposed in literature. The MOMHs were tested on some commonly used benchmark MOPs and/or applied to real-world MOPs. The main challenges to achieve high performance multiobjective optimization include guiding the search towards the true Pareto front and obtaining reasonably distributed nondominated solutions.

Nondominated sorting genetic algorithm II (NSGA-II) [21] and MOEA/D [19] adopt crossover and mutation to evolve the individuals. MOEA/D-DE [25] replaces the crossover operator of MOEA/D with DE to solve MOPs with complicated Pareto sets. MOEA/D-DE updates an individual based on the difference of two individuals selected from the updated individual’s neighborhood, assuming that such a difference provides a good search direction. Gaussian mutation was employed in CMPSO [14] to help refine the externally stored elitists.

The diversity of the elitists can be promoted using techniques such as adaptive grid adopted in Pareto archived evolution strategy (PAES) [26] and PAES2 [27], clustering in strength Pareto evolutionary algorithm (SPEA) [28], crowding distance in NSGA-II [21], fitness sharing in niched Pareto genetic algorithm (NPGA) [29], maximin sorting in [30], *M*-nearest-neighbors product-based vicinity distance in [22] and multiobjective immune algorithm with nondominated neighbor-based selection 2 (NNIA2) [31], nearest neighbor density estimation in SPEA2 [32], and weighting based aggregation in MOEA/D [19].

Hypervolume, introduced by Zitzler and Thiele [28], is a desirable metric used to evaluate the performance of a MOMH, as the maximization of hypervolume constitutes the necessary and sufficient conditions for deriving maximally diverse nondominated solutions over the true Pareto front [33]. Recently, some hypervolume based MOMHs [34–36] have been proposed and they directly use the hypervolume metric to act as a selection pressure rewarding convergence and diversity. However, it is NP-hard to calculate the hypervolume metric [37].

Many MOMHs are decomposition based. The earliest decomposition based MOMH is vector evaluated genetic algorithm (VEGA) [38]. VEPSO [11] is an adaptation of VEGA to the PSO framework. Harrison et al. [39] investigated various strategies for direct information sharing among the swarms in VEPSO. The multigroup learning automata based approach proposed in [40] utilizes a synergistic learning strategy to encourage each group to learn not only from the elitists but also from the search experience of all the other groups. Zhang et al. [41] enhanced the performance of MOEA/D-DE with a dynamic resource allocation strategy. The works [42–47] are other recent improvements on MOEA/D.

## 3. Multiswarm comprehensive learning particle swarm optimization

### 3.1 Basic architecture

**Fig 1:**
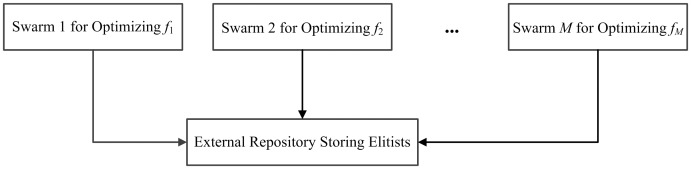
Basic architecture of MSCLPSO.

MSCLPSO is a decomposition based multiswarm MOPSO [23]. As Fig 1 shows, *M* swarms are used and each swarm *m* (1 ≤ *m* ≤ *M*) focuses on optimizing objective *f*_*m*_ using CLPSO. Elitists are stored into an external repository. The repository is shared by all the swarms. Each swarm *m* doesn’t learn from the elitists and the search experience of any other swarm.

### 3.2 Maintenance of the external repository

The external repository **REP** is initialized to be empty. As the number of elitists quickly grows during the run, **REP** has a fixed maximum size *R*_max_. **REP** is maintained as follows in every generation [23]:

Step 1) A temporary set **TMP** is initialized to be empty.
Step 2) All the elitists in **REP** are added into **TMP**.
Step 3) For each particle *i* in each swarm *m*, the particle’s position *P*_*m*,*i*_ is added into **TMP** if *P*_*m*,*i*_ is feasible (i.e. within $\left[\underline{P_{d}},\bar{P_{d}}\right]$ on each dimension *d*).
Step 4) Apply mutation to some elitists of **REP** and add the mutated solutions into **TMP**.
Step 5) Apply DE to a number of extreme and least crowded elitists of **REP** and add the differentially evolved solutions into **TMP**.
Step 6) Set **REP** to be empty.
Step 7) Each solution in **TMP** is checked whether it is dominated by any other solution in **TMP**. Any dominated solution is removed from **TMP**.
Step 8) Sort the remaining elitists in **TMP** in the decreasing order of crowding/vicinity distances. If the number of the elitists in **TMP** is larger than *R*_max_, the first *R*_max_ elitists are allowed to stay in **TMP** and the others are removed from **TMP**. All the elitists in **TMP** are then copied to **REP**.

In Step 8), the elitists in **TMP** are sorted using respectively the crowding distance technique [21] for two-objective MOPs and the *M*-nearest-neighbors product-based vicinity distance technique [22] for MOPs with more than two objectives. The crowding/vicinity distance of an elitist provides an estimate of the density of the surrounding solutions. The crowding distance of an elitist is a weighted distance of the elitist’s two neighboring solutions, while the vicinity distance is the multiplication product of the distances between the elitist and the elitist’s *M* nearest neighbors. Allowing the nondominated solutions with larger crowding/vicinity distances to stay in **REP** enhances the diversity of the resulting elitists on the Pareto front.

### 3.3 Mutation

As briefly discussed in [23], the personal best positions and elitists carry useful information about the Pareto set. The mutation strategy adopted in MSCLPSO exploits the personal best positions and the differences of the elitists. After a sufficient number of generations, the personal best position *B*_*m*,*i*_ of particle *i* in swarm *m* is an exact-optimum or near-optimum corresponding to objective *f*_*m*_. If the Pareto-optimal decision vectors are indifferent on dimension *d*, *B*_*m*,*i*,*d*_ might be close to dimension *d* of the Pareto-optimal decision vector that is optimal on objective *f*_*m*_, hence learning from *B*_*m*,*i*,*d*_ might contribute to the search of the Pareto set on dimension *d*; on the other hand, if the Pareto set is complicated on dimension *d*, the personal best positions obtained by different swarms often differ considerably on that dimension, accordingly learning from the personal best positions leads to the search of different regions of the Pareto set on dimension *d*. In addition, the difference between two different elitists on dimension *d* is often small in the simple cases and could be large in the complicated cases.

To be more specific, let the number of the elitists in **REP** be *R*, the maximum number of mutations be *N*_mut_, and the mutation tradeoff probability be *α*, the details of the mutation strategy in Step 4) of the external repository maintenance procedure are described in the following.

Step 4.1) Initialize the mutation counter *n* = 1.
Step 4.2) If *n* ≤ min{*N*_mut_, *R*}, go to Step 4.3); otherwise, return.
Step 4.3) Randomly select an elitist *l* from **REP**. *l*’s decision vector is copied as *Q*_*l*_ = {*Q*_*l*,1_, *Q*_*l*,2_, …, *Q*_*l*,*D*_}. Randomly select a dimension *d*. Generate a random number *r*_mut1_ uniformly distributed in the range [0, 1]. If *r*_mut1_ < *α* or *R* < 2, go to Step 4.4); otherwise, go Step 4.5).
Step 4.4) Randomly select a swarm *m*. Randomly select a particle *i* in swarm *m*. Mutate *Q*_*l*,*d*_ according to Eq (6).
  $$Q_{l,d}=Q_{l,d}+r_{\text{mut2}}(B_{m,i,d}-Q_{l,d})$$ (6)
  where *r*_mut2_ is a random number uniformly distributed in the range [0, 1].
Step 4.5) Randomly select two different elitists *l*1 and *l*2 from **REP**. *l*1 and *l*2 don’t need to be different from *l*. Mutate *Q*_*l*,*d*_ according to Eq (7).
  $$Q_{l,d}=Q_{l,d}+r_{\text{mut3}}(Z_{l1,d}-Z_{l2,d})$$ (7)
  where *r*_mut3_ is a random number uniformly distributed in the range [0, 1]; and *Z*_*l*1_ and *Z*_*l*2_ are respectively the decision vector of *l*1 and that of *l*2.
Step 4.6) Repair *Q*_*l*,*d*_ using the re-initialization technique introduced in MOEA/D-DE [25, 41] if *Q*_*l*,*d*_ is outside the dimensional search space $\left[\underline{P_{d}},\bar{P_{d}}\right]$.
Step 4.7) Add *Q*_*l*_ into **TMP**.
Step 4.8) Increase the mutation counter *n* = *n* + 1, and go back to Step 4.2).

The mutation tradeoff probability *α* controls whether to mutate based on the personal best position using Eq (6) or the difference of the elitists using Eq (7). *α* is suggested to take a medium value in the range [0, 1] so as to achieve a balanced use of the information embodied in the personal best positions and elitists. The number of maximum mutations *N*_mut_ can be less than *R*_max_. As can be seen from Step 4.3), each elitist and each dimension has an equal chance to be selected.

### 3.4 Differential evolution

Let the maximum number of DEs be *N*_de_, for each elitist *l* in **REP** with 1 ≤ *l* ≤ min{*N*_de_, *R*}, *l*’s decision vector is copied as *Q*_*l*_. Let the DE tradeoff probability be *β*, a random number *r*_de1_ uniformly distributed in the range [0, 1] is generated. If *r*_de1_ < *β* and *R* ≥ 2, each dimension of *Q*_*l*_ is differentially evolved according to Eq (8); otherwise according to Eq (9). All the dimensions use the same pair of DE coefficients *r*_de2_ and *r*_de3_, with *r*_de2_ and *r*_de3_ being two random numbers generated from a normal distribution with mean 0.5 and standard deviation 0.5.

$$Q_{l,d}=\left\{ \begin{array}{l} Q_{l,d}+r_{\text{de2}}(Z_{l1,d}-Q_{l,d})+r_{\text{de3}}(Q_{l,d}-Z_{l2,d}),\text{ if }\Delta_{l1,l}\geq \Delta_{l2,l}\\ Q_{l,d}+r_{\text{de2}}(Z_{l2,d}-Q_{l,d})+r_{\text{de3}}(Q_{l,d}-Z_{l1,d}),\text{ otherwise} \end{array}\right.$$ (8)

$$Q_{l,d}=\left\{ \begin{array}{l} Q_{l,d}+\delta \text{(}\bar{P_{d}}-\underline{P_{d}}),\text{ if }r_{\text{de2}}Z_{l1,d}-r_{\text{de3}}Z_{l2,d}>\delta \text{(}\bar{P_{d}}-\underline{P_{d}}\text{)}\\ Q_{l,d}-\delta \text{(}\bar{P_{d}}-\underline{P_{d}}),\text{ else if }r_{\text{de2}}Z_{l1,d}-r_{\text{de3}}Z_{l2,d}<-\delta \text{(}\bar{P_{d}}-\underline{P_{d}}\text{)}\\ Q_{l,d}+r_{\text{de2}}Z_{l1,d}-r_{\text{de3}}Z_{l2,d},\text{ otherwise} \end{array}\right.$$ (9)

where *l*1 and *l*2 are two elitists randomly selected from **REP**; Δ_*l*1,*l*_ is the Euclidean distance between *l*1 and *l* in the objective space; Δ_*l*2,*l*_ is the Euclidean distance between *l*2 and *l* in the objective space; and *δ* is the velocity limiter which is a number in the range (0, 1]. *Q*_*l*,*d*_ is further repaired using the re-initialization technique introduced in MOEA/D-DE [25, 41] if *Q*_*l*,*d*_ is outside the dimensional search space $\left[\underline{P_{d}},\bar{P_{d}}\right]$. After all the dimensions are differentially evolved, *Q*_*l*_ is added into **TMP**. The above details explain Step 5) of the external repository maintenance procedure. The number of maximum DEs *N*_de_ can be less than *R*_max_.

Remember that in Step 7), the elitists in **TMP** are sorted in the decreasing order of crowding/vicinity distances, and then copied to **REP**. Therefore, the smaller the index of an elitist in **REP**, the larger the crowding/vicinity distance of that elitist. MSCLPSO applies DE to a number of elitists with the smallest indices in **REP**. In other words, the differentially evolved elitists are extreme and least crowded. Note that an elitist that is extreme on a single objective is assigned an infinite crowding/vicinity distance [21, 22]; though such an elitist may actually be crowded, it may however be still far from the corresponding true extreme nondominated solution on the Pareto front. The application of DE to the extreme and least crowded elitists is expected to improve the diversity of the elitists [23].

In Eq (8), *l*1 and *l*2 don’t need to be different, but at least one of them is different from *l*. Eq (8) pushes *l* towards the more distant (measured in the objective space) elitist of *l*1 and *l*2, and in the meanwhile pulls *l* away from the nearer elitist, with the purpose of exploring the search space. In addition, Eq (8) provides more diverse search directions than the DE operators used in literature MOMHs.

Eq (9) also provides diverse search directions. *l*1, *l*2, and *l* don’t need to be mutually different. The term *r*_de2_*Z*_*l*1,*d*_—*r*_de3_*Z*_*l*2,*d*_ is clamped to the range $\left[-\delta (\bar{P_{d}}-\underline{P_{d}}),\delta (\bar{P_{d}}-\underline{P_{d}})\right]$. *δ* is suggested to take a small value in order to facilitate exploiting the region near *l*.

The mutation tradeoff probability *β* thus is suggested to take a medium value to achieve a balance between the exploration and exploitation of the search space. The assumption of the DE strategy is that the Pareto-optimal decision vectors are somewhat correlated and the learning from *l*1 and *l*2 could provide an appropriate search direction for the evolution of *l*.

### 3.5 Flow chart and complexity analysis

**Fig 2:**
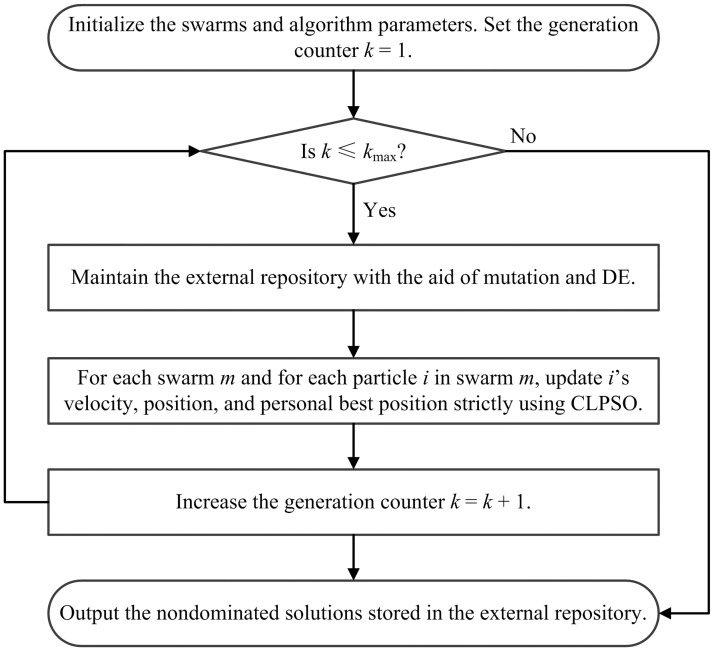
Flow chart of MSCLPSO.

The swarms obtain personal best positions that carry useful information about the Pareto set. The mutation and DE strategies help MSCLPSO discover the true Pareto front. The flow chart of MSCLPSO is depicted in Fig 2.

MSCLPSO needs to store various data structures and algorithm parameters. The largest data structure is **TMP** and it requires O(*MN* + *R*_max_ + *N*_mut_ + *N*_de_) memory space. Hence, the space complexity of MSCLPSO is O(*MN* + *R*_max_ + *N*_mut_ + *N*_de_) plus the space required by the objective functions and constraints.

The maintenance of the external repository in Step 3 mainly involves the dominance checking. The dominance checking compares each solution with every other solution in **TMP** on all the objectives. There are maximally *MN* + *R*_max_ + *N*_mut_ + *N*_de_ solutions in **TMP**. Hence the dominance checking requires O((*MN* + *R*_max_ + *N*_mut_ + *N*_de_)^2^*D*) comparisons. In addition, Step 3 requires O(*N*_mut_ + *N*_de_) function evaluations (FEs). The time requirement of CLPSO is O(*ND*) basic operations plus O(*N*) FEs. As there are *M* swarms, Step 4 thus requires O(*MND*) basic operations plus O(*MN*) FEs. Step 1 is executed once. Accordingly, overall MSCLPSO requires O(*k*_max_(*MN* + *R*_max_ + *N*_mut_ + *N*_de_)^2^*D*) basic operations plus O(*k*_max_(*MN* + *N*_mut_ + *N*_de_)) FEs.

## 4. Experimental studies

### 4.1 Performance metric

The inverted generational distance (IGD) [14, 21, 41] has been widely adopted and strongly recommended as a performance metric for evaluating MOMHs in recent years, as it can reflect both convergence and diversity of the obtained nondominated solutions. Assuming that the true Pareto front **PF** is known and **U** is a set of uniformly distributed points sampled along PF, the IGD metric is calculated according to Eq (10).

$$\frac{\sum_{u}s_{u}}{U}$$ (10)

where *u* is a point in U; *s*_*u*_ is the Euclidean distance between *u* and the nondominated solution in **REP** that is nearest to *u*, measured in the objective space; and *U* is the number of points in U. It is clear that if the nondominated solutions in **REP** have a good spread along the true Pareto front, the IGD value will be small.

### 4.2 Benchmark problems

Various benchmark MOPs have been proposed in literature to evaluate MOMHs. The following benchmark MOPs are chosen: ZDT2 and ZDT3 from the ZDT test set [2], two modified versions of ZDT4 called ZDT4-V1 and ZDT4-V2, WFG1 from the WFG test set [48], UF1, UF2, UF7, UF8, and UF9 from the UF test set [49]. Eqs (11) and (12) respectively describe the ZDT4-V1 and ZDT4-V2 problems.

$$\begin{array}{l} \text{ZDT}4-\text{V}1:\;\left\{ \begin{array}{l} f_{1}=x_{1}\\ f_{2}=y(1-\sqrt{\frac{f_{1}}{y}}) \end{array}\right.,\;\\ \text{where }y=1+10(D-1)+\sum_{d=2}^{D}\left({\left(x_{d}\right)}^{2}-10\text{cos}(4\pi x_{d})\right),\;x_{1}\in [0,1],x_{d}\in [-1,1],2\leq d\leq D \end{array}$$ (11)

$$\begin{array}{l} \text{ZDT}4-\text{V}2:\;\left\{ \begin{array}{l} f_{1}=x_{1}\\ f_{2}=y(1-\sqrt{\frac{f_{1}}{y}}) \end{array}\right.\text{, }\\ \text{where }y=1+10(D-1)+\sum_{d=2}^{D}\left({\left(h_{d}\right)}^{2}-10\text{cos}(4\pi h_{d})\right)\text{, }h_{d}=1000(x_{d}-0.5),x_{1}\in [0,1],x_{d}\in [-5,2],2\leq d\leq D \end{array}$$ (12)

As can be seen from Eqs (11) and (12), *y* is similar to the complex multimodal Rastrigin’s function [50]. ZDT4-V1 is the same with ZDT4 except that the search space of *x*_*d*_ (2 ≤ *d* ≤ *D*) is [-1, 1] in ZDT4-V1 while [-5, 5] in ZDT4. The characteristics of the 10 benchmark MOPs are listed in Table 1. The problems exhibit different characteristics such as 2 and 3 objectives, 10 and 30 dimensions, unimodal and multimodal objective functions, linear, convex, concave, disconnected, and mixed Pareto fronts, and simple and complicated Pareto sets. Therefore, the problems can be used to evaluate the performance of MOMHs from various aspects. In Table 1, *o* denotes the number of position-related working parameters for the WFG1 problem and is set as 2. S1 File gives the source code of the MSCLPSO algorithm with all the benchmark problems.

**Table 1:** Characteristics of all the benchmark problems.

Problem	*M*	*D*	Search space	Pareto set	Pareto front
ZDT2	2	30	*x*_*d*_ ∈ [0, 1], 1 ≤ *d* ≤ *D*	*x*_*1*_ ∈ [0, 1], *x*_*d*_ = 0, 2 ≤ *d* ≤ *D*	Concave, *f*_1_ U*, *f*_2_ M**
ZDT3	2	30	*x*_*d*_ ∈ [0, 1], 1 ≤ *d* ≤ *D*	*x*_*1*_ ∈ [0, 1], *x*_*d*_ = 0, 2 ≤ *d* ≤ *D*	Convex, disconnected, *f*_1_ U, *f*_2_ M
ZDT4-V1	2	10	*x*_*1*_ ∈ [0, 1], *x*_*d*_ ∈ [-1, 1], 2 ≤ *d* ≤ *D*	*x*_*1*_ ∈ [0, 1], *x*_*d*_ = 0, 2 ≤ *d* ≤ *D*	Convex, *f*_1_ U, *f*_2_ M
ZDT4-V2	2	10	*x*_*1*_ ∈ [0, 1], *x*_*d*_ ∈ [-5, 2], 2 ≤ *d* ≤ *D*	*x*_*1*_ ∈ [0, 1], *x*_*d*_ = 0.5, 2 ≤ *d* ≤ *D*	Convex, *f*_1_ U, *f*_2_ M
WFG1	2	10	*x*_*d*_ ∈ [0, 2*d*], 1 ≤ *d* ≤ *D*	*x*_*d*_ ∈ [0, 2*d*], 1 ≤ *d* ≤*o*, *x*_*d*_ = 0.35, *o* + 1 ≤ *d* ≤*D*	Convex, mixed, *f*_1_ U, *f*_2_ U
UF1	2	30	*x*_*1*_ ∈ [0, 1], *x*_*d*_ ∈ [-1, 1], 2 ≤ *d* ≤ *D*	$\begin{array}{l} x_{1}\in [0,1],\\ x_{d}=\text{sin}(6\pi x_{1}+\frac{d\pi }{D}),2\leq d\leq D \end{array}$	Convex, *f*_1_ M, *f*_2_ M
UF2	2	30	*x*_*1*_ ∈ [0, 1], *x*_*d*_ ∈ [-1, 1], 2 ≤ *d* ≤ *D*	$\begin{array}{l} x_{1}\in [0,1],\\ x_{d}=(0.3{\left(x_{1}\right)}^{2}\text{cos}(24\pi x_{1}+\frac{4d\pi }{D})+0.6x_{1})\\ \text{cos}(6\pi x_{1}+\frac{d\pi }{D}),\text{ if 2}\leq d\leq D\text{ and }d\text{ is odd},\\ x_{d}=(0.3{\left(x_{1}\right)}^{2}\text{cos}(24\pi x_{1}+\frac{4d\pi }{D})+0.6x_{1})\\ \text{sin}(6\pi x_{1}+\frac{d\pi }{D}),\text{ if 2}\leq d\leq D\text{ and }d\text{ is even} \end{array}$	Convex, *f*_1_ M, *f*_2_ M
UF7	2	30	*x*_*1*_ ∈ [0, 1], *x*_*d*_ ∈ [-1, 1], 2 ≤ *d* ≤ *D*	$\begin{array}{l} x_{1}\in [0,1],\\ x_{d}=\text{sin}(6\pi x_{1}+\frac{d\pi }{D}),2\leq d\leq D \end{array}$	Linear, *f*_1_ M, *f*_2_ M
UF8	3	30	*x*_*1*_ ∈ [0, 1], *x*_*2*_ ∈ [0, 1], *x*_*d*_ ∈ [-2, 2], 3 ≤ *d* ≤ *D*	$\begin{array}{l} x_{1}\in [0,1],x_{2}\in [0,1],\\ x_{d}=2x_{2}\text{sin}(2\pi x_{1}+\frac{d\pi }{D}),3\leq d\leq D \end{array}$	Concave, *f*_1_ M, *f*_2_ M, *f*_3_ M
UF9	3	30	*x*_*1*_ ∈ [0, 1], *x*_*2*_ ∈ [0, 1], *x*_*d*_ ∈ [-2, 2], 3 ≤ *d* ≤*D*	$\begin{array}{l} x_{1}\in [0,0.25]\cup [0.75,1],x_{2}\in [0,1],\\ x_{d}=2x_{2}\text{sin}(2\pi x_{1}+\frac{d\pi }{D}),3\leq d\leq D \end{array}$	Linear, disconnected, *f*_1_ M, *f*_2_ M, *f*_3_ M

* U refers to unimodal.

** M refers to multimodal.

### 4.3 MOMHs compared and parameter settings

Two performance issues are investigated: 1) can the personal best positions, mutation, and DE help MSCLPSO discover the true Pareto front? and 2) how does MSCLPSO perform compared with other literature MOMHs? For the first issue, two MSCLPSO variants, namely MSCLPSO-1 and MSCLPSO-2, are studied. In MSCLPSO-1, the mutation tradeoff probability *α* = 0, i.e. the mutation strategy doesn’t learn from the personal best positions. In MSCLPSO-2, the maximum number of DEs *N*_de_ = 0, i.e. the DE strategy is not invoked. Concerning the second issue, MSCLPSO is compared with four representative MOMHs: CMPSO [14], MOEA/D [19], MOEA/D-DE [41], and NSGA-II [21].

The parameters of the MSCLPSO variants are determined based on trials on all the benchmark MOPs and are listed in Table 2. The parameters of CLPSO take the recommended values stated in Subsection 2.1. The parameters of CMPSO, MOEA/D, and NSGA-II take the recommended values given in the corresponding references. The elitists are externally stored in a repository for the MSCLPSO variants and CMPSO and internally preserved in the evolving population for MOEA/D and NSGA-II. To facilitate fair comparison, the (maximum) number of externally/internally stored elitists is set as 100 on the 2-objective benchmark MOPs and 300 on the 3-objective MOPs. *U* is set as 1000 and 10000 respectively for the 2- and 3-objective MOPs. The benchmark MOPs require different number of FEs to obtain the shape of the true Pareto front due to their different difficulty levels. The FEs values on the problems are listed in Table 3. *ε*-dominance is applied to the MSCLPSO variants and CMPSO and *ε* = 0.0001. The MSCLPSO variants, CMPSO, MOEA/D, and NSGA-II are executed for 30 independent runs on each problem.

**Table 2:** Algorithm parameters of the MSCLPSO variants.

MSCLPSO variant	Parameters
MSCLPSO	*N* = 10, *α* = 0.5, *β* = 0.5, *δ* = 5%, *N*_mut_ = *R*_max_ (*M*– 1) / 5, *N*_de_ = *R*_max_ (*M*– 1) / 10
MSCLPSO-1	*N* = 10, *α* = 0, *β* = 0.5, *δ* = 5%, *N*_mut_ = *R*_max_ (*M*– 1) / 5, *N*_de_ = *R*_max_ (*M*– 1) / 10
MSCLPSO-2	*N* = 10, *α* = 0.5, *β* = 0.5, *δ* = 5%, *N*_mut_ = 3 *R*_max_ (*M*– 1) / 10, *N*_de_ = 0

**Table 3:** Number of function evaluations on all the benchmark problems.

Problem	ZDT2	ZDT3	ZDT4-V1	ZDT4-V2	WFG1	UF1	UF2	UF7	UF8	UF9
FEs	10E4	10E4	10E4	25E4	100E4	35E4	100E4	40E4	90E4	90E4

In [41], MOEA/D-DE was evaluated on the UF problems with complicated Pareto sets. MOEA/D-DE used a population of 600 individuals to solve the 2-objective UF problems and a population of 1000 individuals to solve the 3-objective UF problems. 100 elitists and 150 elitists were selected from the final population to calculate the IGD metric respectively on the 2- and 3-objective problems. In contrast, MOEA/D used populations of 100 and 300 individuals respectively on the 2-objective ZDT and 3-objective DTLZ problems with simple Pareto sets in [19]. It seems like MOEA/D-DE doesn’t have a unified parameter setting framework for various benchmark MOPs. Therefore, MSCLPSO is compared with MOEA/D-DE only on the UF1, UF2, UF7, UF8, and UF9 problems with 300000 FEs on each of the problems. The performance data of MOEA/D-DE are directly copied from [41]. On the UF8 and UF9 problems, 150 elitists with the largest vicinity distances are selected from the final external repository of MSCLPSO.

### 4.4 Experimental results and discussions

**Fig 3:**
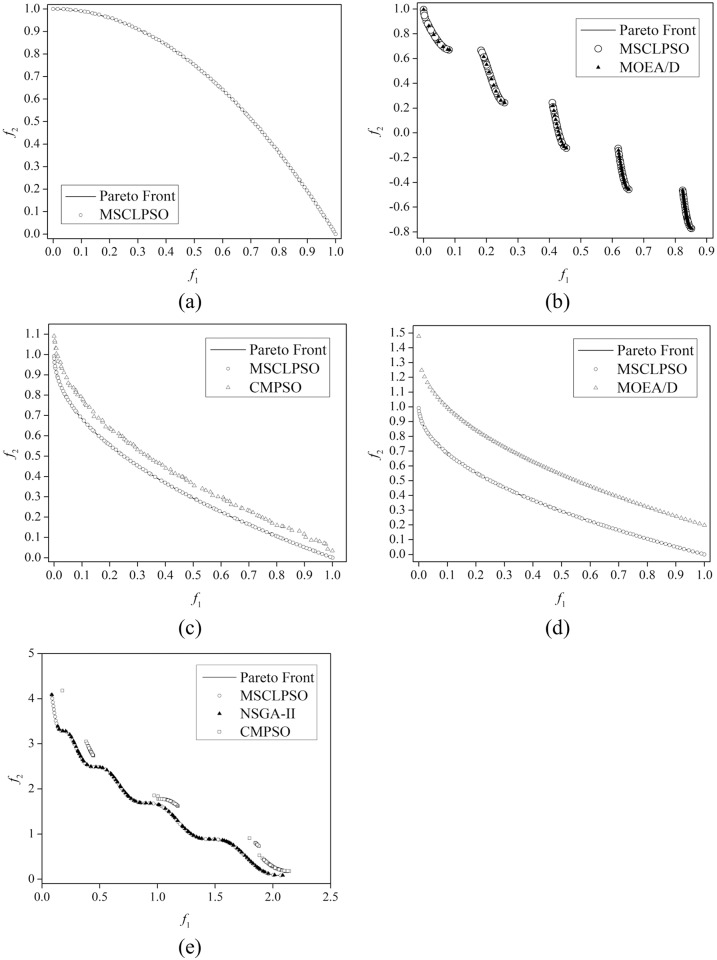
Final nondominated solutions obtained on the ZDT and WFG benchmark problems. (a) MSCLPSO in the best run on ZDT2 (b) MSCLPSO in the best run and MOEA/D in the best run on ZDT3 (c) MSCLPSO in the best run and CMPSO in the worst run on ZDT4-V1 (d) MSCLPSO in the best run and MOEA/D in the best run on ZDT4-V2 (e) MSCLPSO in the best run, NSGA-II in the best run, and CMPSO in the best run on WFG1.

**Fig 4:**
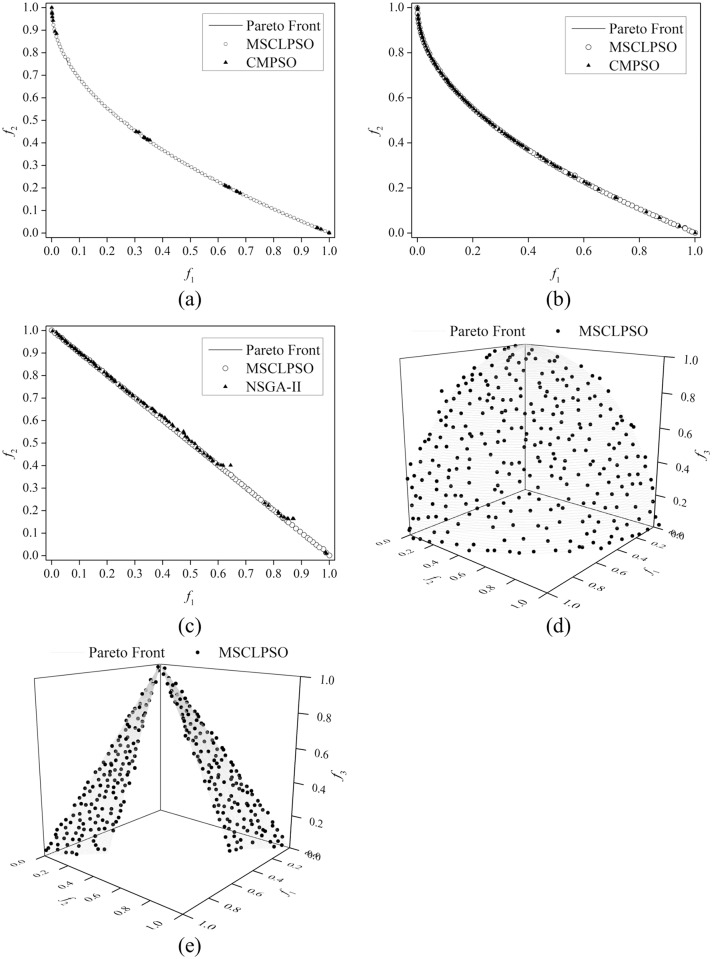
Final nondominated solutions obtained on the UF benchmark problems. (a) MSCLPSO in the best run and CMPSO in the best run on UF1 (b) MSCLPSO in the best run and CMPSO in the best run on UF2 (c) MSCLPSO in the best run and NSGA-II in the best run on UF7 (d) MSCLPSO in the best run on UF8 (e) MSCLPSO in the best run on UF9.

Table 4 lists the IGD results related to the 30 runs of the MSCLPSO variants, CMPSO, MOEA/D, and NSGA-II on all the benchmark MOPs. MSCLPSO, CMPSO, MOEA/D, and NSGA-II are ranked according to their mean IGD results and the MOMHs are compared using the wellknown Wilcoxon rank sum test with the significance level 0.05. The ranking and Wilcoxon rank sum test results are listed in Table 5. The final single-objective best solutions obtained by the swarms of MSCLPSO on all the benchmark MOPs are listed in Table 6. Table 7 compares MSCLPSO and MOEA/D-DE on the UF1, UF2, UF7, UF8, and UF9 problems. Table 8 gives the IGD results of MSCLPSO using some different parameter settings. In Tables 4 and 7, the best results on each problem are marked in bold. The final nondominated solutions obtained by MSCLPSO and some literature MOMHs on all the benchmark MOPs are illustrated in Figs 3 and 4. The data set underlying Figs 3 and 4 can be found in S2 File.

**Table 4:** IGD results of the MSCLPSO variants, CMPSO, MOEA/D, and NSGA-II on all the benchmark problems.

Problem	IGD result	MSCLPSO	MSCLPSO-1	MSCLPSO-2	CMPSO	MOEA/D	NSGA-II
ZDT2	Mean	4.35E-3	4.38E-3	7.95E-3	4.14E-3	**3.80E-3**	4.81E-3
	SD	1.08E-4	9.79E-5	1.06E-3	1.01E-4	**1.53E-7**	2.07E-4
	Best	4.11E-3	4.20E-3	6.18E-3	3.99E-3	**3.80E-3**	4.49E-3
	Worst	4.57E-3	4.54E-3	1.02E-2	4.39E-3	**3.80E-3**	5.37E-3
ZDT3	Mean	**4.87E-3**	4.89E-3	5.55E-3	6.35E-3	8.77E-3	5.51E-3
	SD	9.41E-5	1.04E-4	2.12E-4	8.43E-4	**1.88E-5**	2.38E-4
	Best	**4.72E-3**	4.76E-3	5.10E-3	5.31E-3	8.75E-3	5.10E-3
	Worst	**5.07E-3**	5.21E-3	6.01E-3	8.93E-3	8.86E-3	6.15E-3
ZDT4-V1	Mean	**4.38E-3**	3.16E-2	8.19E-3	1.16E-2	7.81E-2	4.18E-2
	SD	**1.25E-4**	6.05E-2	2.22E-2	1.04E-2	9.02E-2	8.12E-2
	Best	4.19E-3	4.21E-3	3.97E-3	4.30E-3	**3.87E-3**	4.36E-3
	Worst	**4.81E-3**	2.56E-1	1.26E-1	5.13E-2	2.56E-1	2.56E-1
ZDT4-V2	Mean	**4.26E-3**	3.30E-2	8.20E-3	3.67	7.37E-1	6.60E-1
	SD	**1.04E-4**	6.23E-2	2.22E-2	2.44	3.85E-1	3.51E-1
	Best	4.04E-3	4.13E-3	**3.94E-3**	9.59E-1	1.86E-1	2.58E-1
	Worst	**4.59E-3**	2.56E-1	1.26E-1	9.93	1.71	1.60
WFG1	Mean	**1.35E-2**	1.39E-2	1.38E-2	5.48E-1	7.72E-1	2.53E-1
	SD	**4.11E-4**	5.57E-4	4.94E-4	2.10E-1	2.53E-2	1.38E-1
	Best	**1.29E-2**	1.31E-2	1.29E-2	2.57E-1	7.29E-1	3.04E-2
	Worst	**1.45E-2**	1.52E-2	1.49E-2	9.16E-1	8.18E-1	5.60E-1
UF1	Mean	**4.26E-3**	4.26E-3	3.10E-2	6.29E-2	1.28E-1	7.68E-2
	SD	1.35E-4	**1.02E-4**	1.19E-2	1.59E-2	6.96E-2	2.07E-2
	Best	**4.08E-3**	4.13E-3	1.37E-2	4.02E-2	4.36E-2	5.37E-2
	Worst	4.79E-3	**4.50E-3**	6.04E-2	9.76E-2	2.70E-1	1.48E-1
UF2	Mean	4.42E-3	**4.34E-3**	1.02E-2	1.60E-2	2.98E-2	2.69E-2
	SD	2.14E-4	**1.95E-4**	2.33E-3	3.14E-3	2.24E-2	1.05E-2
	Best	4.17E-3	**4.03E-3**	7.01E-3	1.05E-2	1.19E-2	1.53E-2
	Worst	4.99E-3	**4.96E-3**	1.83E-2	2.55E-2	1.20E-1	5.73E-2
UF7	Mean	**4.30E-3**	4.33E-3	4.98E-2	1.04E-1	4.24E-1	7.32E-2
	SD	**1.17E-4**	1.29E-4	8.25E-2	1.04E-1	1.68E-1	1.15E-1
	Best	4.09E-3	**4.08E-3**	1.01E-2	3.80E-2	4.03E-2	1.99E-2
	Worst	4.64E-3	**4.56E-3**	2.91E-1	3.74E-1	6.45E-1	4.26E-1
UF8	Mean	**4.75E-2**	4.95E-2	1.16E-1	3.94E-1	1.88E-1	4.00E-1
	SD	**3.22E-3**	1.16E-2	4.06E-2	3.83E-2	1.68E-1	2.84E-2
	Best	**4.28E-2**	4.11E-2	7.32E-2	2.58E-1	8.23E-2	3.20E-1
	Worst	**5.44E-2**	1.07E-1	2.42E-1	4.30E-1	7.49E-1	4.22E-1
UF9	Mean	**2.70E-2**	6.48E-2	3.85E-2	9.38E-2	1.59E-1	3.33E-1
	SD	**1.58E-3**	6.62E-2	9.73E-3	3.44E-2	2.98E-2	2.77E-2
	Best	**2.37E-2**	2.45E-2	2.52E-2	5.63E-2	4.99E-2	2.91E-1
	Worst	**3.01E-2**	2.08E-1	6.74E-2	2.06E-1	1.90E-1	3.73E-1

**Table 5:** Ranks of MSCLPSO, CMPSO, MOEA/D, and NSGA-II in term of the mean IGD results on all the benchmark problems.

Problem	ZDT2	ZDT3	ZDT4-V1	ZDT4-V2	WFG1	UF1	UF2	UF7	UF8	UF9	Total	Rank
MSCLPSO	3	1	1	1	1	1	1	1	1	1	12	1
CMPSO	2^+^	3^-^	2^-^	4^-^	3^-^	2^-^	2^-^	3^-^	3^-^	2^-^	26	2
MOEA/D	1^+^	4^-^	4^-^	3^-^	4^-^	4^-^	4^-^	4^-^	2^-^	3^-^	33	4
NSGA-II	4^-^	2^-^	3^-^	2^-^	2^-^	3^-^	3^-^	2^-^	4^-^	4^-^	29	3

^+^ The corresponding MOMH is significantly better than MSCLPSO according to the Wilcoxon rank sum test.

^-^ The corresponding MOMH is significantly worse than MSCLPSO according to the Wilcoxon rank sum test.

**Table 6:** Final single-objective best solutions obtained by the swarms of MSCLPSO on all the benchmark problems.

Problem	Swarm 1		Swarm 2		Swarm 3	
	Mean	SD	Mean	SD	Mean	SD
ZDT2	4.30E-20	1.80E-19	4.95E-2	2.23E-2	-	-
ZDT3	3.82E-11	2.09E-10	-7.60E-1	6.25E-3	-	-
ZDT4-V1	3.40E-23	1.81E-22	6.67E-2	8.48E-2	-	-
ZDT4-V2	1.10E-29	6.03E-29	3.55E-2	7.00E-2	-	-
WFG1	1.04E-1	9.85E-3	8.30E-2	1.41E-17	-	-
UF1	3.02E-2	2.99E-2	2.29E-2	2.68E-2	-	-
UF2	2.23E-16	8.31E-16	1.49E-2	1.88E-2	-	-
UF7	9.90E-2	2.08E-1	1.55E-2	1.17E-2	-	-
UF8	1.06E-3	3.34E-3	1.09E-5	2.18E-5	5.74E-5	1.12E-4
UF9	3.65E-5	7.49E-5	6.34E-4	2.38E-3	2.52E-2	4.68E-2

**Table 7:** Comparison of MSCLPSO and MOEA/D-DE on the UF benchmark problems.

		UF1	UF2	UF7	UF8	UF9
MSCLPSO	Mean	4.40E-3	**6.19E-3**	4.62E-3	9.29E-2	**5.07E-2**
	SD	**1.83E-4**	**8.24E-4**	**3.21E-4**	3.45E-2	**2.59E-2**
	Best	4.16E-3	5.05E-3	4.25E-3	6.18E-2	3.73E-2
	Worst	**5.10E-3**	**8.99E-3**	**5.60E-3**	2.04E-1	1.84E-1
MOEA/D-DE	Mean	**4.35E-3**	6.79E-3	**4.44E-3**	**5.84E-2**	7.90E-2
	SD	2.90E-4	1.82E-3	1.17E-3	**3.21E-3**	5.42E-2
	Best	**3.99E-3**	**4.81E-3**	**4.05E-3**	**5.07E-2**	**3.50E-2**
	Worst	5.19E-3	1.09E-2	1.06E-2	**6.56E-2**	**1.50E-1**

**Table 8:** IGD results of MSCLPSO using some different parameter settings.

Parameter setting	Problem	IGD result			
		Mean	SD	Best	Worst
*α* = 1	UF1	5.44E-3	5.40E-4	4.87E-3	7.20E-3
*β* = 0	UF1	6.40E-3	5.21E-3	4.29E-3	3.13E-2
*β* = 1	UF7	4.49E-3	3.88E-4	4.04E-3	5.70E-3
*δ* = 1%	UF7	4.75E-3	3.58E-4	4.24E-3	5.57E-3
*δ* = 10%	ZDT2	4.41E-3	1.15E-4	4.16E-3	4.68E-3
*δ* = 20%	UF8	5.04E-2	1.81E-2	4.16E-2	1.44E-1

#### The personal best positions and the mutation strategy

As can be observed from the IGD results given in Table 4 and the final nondominated solutions illustrated in Figs 3 and 4, MSCLPSO can find diverse nondominated solutions reasonably distributed over the true Pareto front on all the 10 benchmark MOPs. The performance of MSCLPSO is rather robust, as indicated from the mean, standard deviation, best, and worst IGD results of MSCLPSO on all the problems. Compared with MSCLPSO, MSCLPSO-1 cannot approximate the true Pareto front on the ZDT4-V1, ZDT4-V2, UF8, and UF9 problems in some runs, as indicated from the worst IGD results of MSCLPSO-1. ZDT4-V1 and ZDT4-V2 have simple Pareto sets, while UF8 and UF9 feature complicated Pareto sets. The final single-objective solutions given in Table 6 show that MSCLPSO can derive an exact-optimum or near-optimum for each single objective on all the 10 benchmark MOPs. The personal best positions obtained by MSCLPSO on objective *f*_2_ on ZDT2, ZDT3, ZDT4-V1, and ZDT4-V2 are close to the Pareto-optimal decision vectors on most dimensions in the search space. The personal best positions obtained by MSCLPSO on both objective *f*_1_ and objective *f*_2_ on WFG1 are close to the Pareto set. The personal best positions obtained by MSCLPSO on different single objectives on UF8 and UF9 are located in rather different regions of the search space. All the observations verify that: 1) CLPSO, owing to its powerful exploration capability, is a proper choice to be adopted in MSCLPSO to help find the personal best positions; 2) the personal best positions carry useful information about the Pareto set, whether the Pareto-optimal decision vectors in the Pareto set are indifferent or significantly different on a dimension; and 3) learning from the personal best position in the mutation strategy benefits the discovery of the true Pareto front.

#### The DE strategy

The IGD results given in Table 4 show that MSCLPSO-2 cannot approximate the true Pareto front on the ZDT4-V1, ZDT4-V2, UF1, UF2, UF7, UF8, and UF9 problems in some or all of the runs. The comparison of MSCLPSO and MSCLPSO-2 indicates that the DE strategy, through leveraging the useful information carried by the elitists, evolves the elitists and is able to explore diverse regions of the search space. The comparison of MSCLPSO, MSCLPSO-1, and MSCLPSO-2 further demonstrates that the combined use of the personal best positions, the mutation strategy, and the DE strategy is required to achieve high performance multiobjective optimization.

#### Comparison of MSCLPSO with CMPSO, MOEA/D, and NSGA-II

As the IGD results given in Table 4 show, CMPSO, MOEA/D, and NSGA-II cannot approximate the true Pareto front on the ZDT4-V1, ZDT4-V2, WFG1, UF1, UF2, UF7, UF8, and UF9 problems in some or all of the runs. MOEA/D performs the best on ZDT2. As can be seen from Fig 3(b), the final nondominated solutions obtained by MOEA/D are not reasonably distributed on the true Pareto front of ZDT3. As Fig 3(c), 3(d) and 3(e) show, CMPSO sometimes gets stuck in a local Pareto front on ZDT4-V1; MOEA/D gets trapped in a local Pareto front even in the best run on ZDT4-V2; CMPSO encounters a local Pareto front in the best run on WFG1, and CMPSO even cannot approximate the entire local Pareto front on WFG1; and NSGA-II can only locate part of the true Pareto front in the best run on WFG1. As indicated from Fig 4(a), 4(b) and 4(c), the MOMHs other than MSCLPSO cannot discover the entire true Pareto on the UF1, UF2, and UF7 problems. Looking at the ranking results given in Table 5, MSCLPSO significantly beats CMPSO, MOEA/D, and NSGA-II on 9 out of the 10 benchmark MOPs and is overall ranked as the best MOMH. All the observations again verify the strengths of the novel techniques adopted in MSCLPSO.

#### Comparison of MSCLPSO and MOEA/D-DE

As can be seen from the IGD results given in Table 7, MSCLPSO ties with MOEA/D-DE in performance on the UF1, UF2, UF7, UF8, and UF9 problems. MOEA/D-DE cannot effectively solve the ZDT4-V1, ZDT4-V2, and WFG1 problems, because: 1) objective *f*_2_ of ZDT4-V1 and ZDT4-V2 is complex multimodal; 2) the Pareto-optimal decision vectors corresponding to WFG1 are not clearly correlated on dimension 1 and dimension 2; and 3) DE often fails in the aforementioned two cases. MSCLPSO is advantageous than MOEA/D-DE in the following aspects: 1) MSCLPSO provides a unified parameter setting framework; 2) MOEA/D-DE needs to determine weight vectors with the largest distances from 5000 randomly selected weight vectors [41]; 3) MOEA/D-DE uses considerably more individuals than MSCLPSO, e.g. MOEA/D-DE uses 600 individuals on the 2-objective UF problems, whereas MSCLPSO just uses 150 individuals in total (with 20 particles, 100 externally stored elitists, 20 individuals for mutation, and 10 individuals for DE); and 4) MOEA/D-DE requires a nontrivial procedure to select nondominated solutions from the final population [41].

#### Tuning of the algorithm parameters

As can be observed from the IGD results given in Table 8, the performance of MSCLPSO is sensitive to the values of the algorithm parameters. The appropriate values of the parameters are determined based on trials on all the benchmark MOPs. *α* = 0 is inappropriate as indicated from the performance data of MSCLPSO-1 given in Table 4, while *α* = 1 is also inappropriate as can be seen from Table 8. Table 8 also shows that *β* = 0 and *β* = 1 are both inappropriate, and *δ* needs to take an appropriate value. The observations demonstrate that: 1) the mutation strategy needs to exploit both the personal best positions and the elitists; and 2) the DE strategy needs to make a tradeoff between exploration and exploitation.

## 5. Conclusions

A metaheuristic called MSCLPSO has been proposed in this paper to achieve high performance multiobjective optimization. MSCLPSO involves multiple swarms, with each swarm focusing on optimizing a separate original objective strictly using the state-of-the-art powerful single-objective metaheuristic CLPSO. Elitists are stored externally. Each swarm doesn’t learn from the elitists and any other swarm. Each particle’s personal best position is determined based on the corresponding single objective, instead of Pareto dominance. MSCLPSO adopts a novel mutation strategy and a novel DE strategy to evolve the elitists. The mutation strategy appropriately exploits the personal best positions and elitists. The DE strategy achieves a balance between exploration and exploitation. MSCLPSO offers a novel technical route to handle multiobjective optimization different from those of existing literature MOMHs. Experiments conducted on various benchmark MOPs have demonstrated that MSCLPSO can robustly derive diverse nondominated solutions distributed reasonably over the true Pareto front in a single run.

## Supporting information

S1 File Java Source Code of the MSCLPSO Algorithm with the Benchmark Problems. (JAVA)

S2 File Origin Data Set for Figs 3 and 4. (OPJ)
